# Coronary Microvascular Dysfunction and Risk of Cardiovascular Events in Type 2 Diabetes Without Obstructive Coronary Artery Disease (CAD): A Prospective Study

**DOI:** 10.7759/cureus.91092

**Published:** 2025-08-27

**Authors:** Umair Abrar, Marium Nadeem Khan, Shafaq Farooq, Hamza Wakil, Hamza Azhar Ghauri, Mariya Qudsia Khanam Sherwani, Ihtisham Akbar, Hamza Ali, Rameez Akhtar, Ali Raza

**Affiliations:** 1 Interventional Cardiology, Department of Cardiovascular Disease, Orthopaedic and Medical Institute (Pvt) Ltd Karachi, Karachi, PAK; 2 Department of Medicine, Shifa International Hospital Islamabad, Islamabad, PAK; 3 Gastroenterology, Holy Family Hospital, Rawalpindi, PAK; 4 Gastroenterology and Hepatology, Royal Alexandra Hospital, NHS Greater Glasgow and Clyde, Paisley, GBR; 5 Interventional Cardiology, Rawalpindi Institute of Cardiology, Rawalpindi, PAK; 6 Department of Orthopedics, Dr. Faisal Masood Teaching Hospital, Sargodha, PAK; 7 Department of Medicine B Ward, DHQ Teaching Hospital, Kohat, PAK; 8 Department of Medicine, Bannu Medical College, Bannu, PAK; 9 Cardiology and Diabetology, Saidu Group of Teaching Hospitals Swat, Swat, PAK; 10 Cardiology, Khyber Teaching Hospital MTI, Peshawar, PAK

**Keywords:** coronary flow reserve, coronary microvascular dysfunction, major adverse cardiovascular events, transthoracic doppler echocardiography, type 2 diabetes mellitus

## Abstract

Background: Coronary microvascular dysfunction (CMD) is increasingly recognized as a contributor to adverse cardiovascular outcomes in patients with diabetes mellitus (DM), yet it remains underdiagnosed. This is largely because routine evaluation is limited by the need for complex, time-consuming, and not routinely performed diagnostic methods, which primarily focus on macrovascular disease.

Objective: To prospectively evaluate the association between CMD and major adverse cardiovascular events (MACE) - defined as myocardial infarction, hospitalization for heart failure, and cardiovascular death - in patients with type 2 diabetes without obstructive coronary artery disease (CAD).

Methodology: We conducted a prospective observational study at Shifa College of Medicine, Islamabad, from August 2022 to July 2024. A total of 264 adults with type 2 DM of five or more years’ duration and non-obstructive CAD (<50% stenosis on angiography/CT) were consecutively enrolled. Exclusion criteria included type 1 diabetes, left ventricular ejection fraction <50%, significant structural or valvular heart disease, prior revascularization, acute coronary syndrome at baseline, and incomplete data. CMD was diagnosed using transthoracic Doppler echocardiography, with coronary flow reserve (CFR) <2.0 considered abnormal in accordance with current consensus. CFR was measured in the left anterior descending artery by two independent operators blinded to outcomes, with reproducibility assessed in a subset. Patients were followed for 24 months; loss to follow-up (7.95%) was excluded from survival analyses. Multivariate Cox regression adjusting for age, sex, hypertension, dyslipidemia, BMI, and diabetes duration was performed, with hazard ratios (HR) and 95% confidence intervals (CI) reported.

Results: Among 264 patients (mean age 58.0 ± 8.1 years, 56.8% male), 142 (53.8%) had CMD. Follow-up was completed in 243 patients (CMD: 128, non-CMD: 115). CMD patients experienced significantly more MACE (29.7% vs. 10.4%, *p*<0.001). On multivariate analysis, CMD remained an independent predictor of MACE (HR 2.41, 95% CI 1.39-4.16, *p*=0.002), myocardial infarction (HR 2.28, 95% CI 1.01-5.16, *p*=0.047), and heart failure hospitalization (HR 2.85, 95% CI 1.19-6.80, *p*=0.018). Cardiovascular mortality was higher in CMD (7.8% vs. 2.6%), while non-cardiovascular mortality was similar between groups. Event-free survival was significantly shorter in CMD patients on Kaplan-Meier analysis.

Conclusion: CMD strongly and independently predicts long-term adverse cardiovascular outcomes in patients with type 2 diabetes without obstructive CAD, even after adjusting for conventional risk factors such as hypertension and smoking. Early detection of CMD using CFR assessment may improve risk stratification and guide preventive management in this high-risk population.

## Introduction

Diabetes mellitus (DM) is a global health challenge and a major risk factor for cardiovascular disease (CVD), which remains the leading cause of morbidity and mortality in diabetic populations [1,2]. Epidemiological studies estimate that 40-50% of patients with diabetes exhibit some degree of coronary microvascular dysfunction (CMD), and recent meta-analyses have shown CMD to be independently associated with nearly a twofold higher risk of major adverse cardiovascular events (MACE) [3-5]. The burden may be even greater in low- and middle-income countries (LMICs), where the prevalence of diabetes is rapidly increasing but access to advanced diagnostic modalities is limited, making CMD an underrecognized contributor to adverse outcomes [6].

CMD is pathophysiologically distinct from diabetic cardiomyopathy: while diabetic cardiomyopathy is primarily characterized by myocardial fibrosis, hypertrophy, and impaired relaxation, CMD reflects impaired regulation of coronary microcirculatory flow and reduced coronary flow reserve (CFR), resulting in ischemia despite angiographically normal epicardial arteries [7,8]. Chronic hyperglycemia, insulin resistance, oxidative stress, and endothelial dysfunction drive these microvascular changes, predisposing to ischemia, heart failure, and mortality [9]. Importantly, CMD has been shown to act as an independent predictor of cardiovascular events beyond traditional risk factors such as hypertension, dyslipidemia, and smoking [10].

Recent improvements in non-invasive imaging techniques, including positron emission tomography (PET), cardiac magnetic resonance (CMR), and transthoracic Doppler echocardiography (TTDE), have made it possible to measure CFR, a critical marker of CMD [11,12]. In this study, TTDE was chosen due to its feasibility, lower cost, and wider availability in LMIC settings compared with PET and CMR. TTDE has been validated in diabetic cohorts, with reported sensitivity ~80% and specificity ~85% for impaired CFR compared to PET. While less precise than advanced imaging, TTDE remains a reliable and accessible method for prospective CMD studies in resource-constrained environments. Studies consistently show that reduced CFR assessed by TTDE is associated with increased risk of myocardial infarction, heart failure hospitalization, and all-cause mortality [13].

Despite growing recognition of CMD, there remains a paucity of prospective outcome data from LMICs, where both DM and CVD are highly prevalent. Most prior studies are from Western populations, often with small sample sizes or short follow-up, leaving a major knowledge gap on CMD’s prognostic role in diabetics in resource-limited contexts. This study was therefore designed to prospectively evaluate whether CMD predicts long-term cardiovascular outcomes in patients with type 2 DM without obstructive coronary artery disease (CAD), providing novel outcome data from a low- to middle-income country setting.

## Materials and methods

Study design and setting

A prospective observational study was conducted at Shifa College of Medicine, Islamabad, from August 2022 to July 2024 to evaluate the association between coronary microvascular dysfunction and long-term cardiovascular outcomes in patients with type 2 diabetes.

Inclusion and exclusion criteria

Adult patients aged ≥40 years with a confirmed diagnosis of T2DM of at least five years’ duration were enrolled, ensuring adequate exposure to chronic hyperglycemia and associated cardiovascular risk. Eligible participants had no evidence of obstructive CAD, defined as <50% epicardial stenosis confirmed by invasive coronary angiography or coronary CT angiography (CTCA) systematically performed in all patients within three months prior to enrollment. Both asymptomatic patients and those presenting with angina-like chest pain or exertional dyspnea were included, provided they had no significant epicardial disease.

Exclusion criteria included type 1 diabetes, left ventricular ejection fraction (LVEF) <50%, significant structural or valvular heart disease, prior coronary revascularization (percutaneous coronary intervention (PCI) or coronary artery bypass grafting (CABG)), acute coronary syndrome at the time of enrollment, or incomplete baseline or follow-up data. All participants underwent TTDE for CFR measurement, and written informed consent was obtained prior to study entry.

Sample size

Using a non-probability convenience sampling method, 264 patients were enrolled. The rationale was to include all consecutive eligible patients at a single center over the study period. While this approach may introduce referral bias, the final sample size is comparable to prior real-world observational studies evaluating CMD and outcomes [14,15]. This limitation is acknowledged in the Discussion.

Data collection

At enrollment, baseline data were comprehensively recorded, including demographics, duration of diabetes, hemoglobin A1C (HbA1C), blood pressure, lipid profile, body mass index (BMI), smoking status, and detailed medication history (statins, angiotensin-converting enzyme inhibitors (ACEi)/angiotensin receptor blockers (ARBs), beta-blockers, sodium-glucose cotransporter 2 (SGLT2) inhibitors, insulin, metformin, and other glucose-lowering agents).

TTDE protocol and CFR cutoff

Coronary flow reserve was assessed in the left anterior descending (LAD) artery using a high-frequency phased-array transducer on the GE Vivid E95 ultrasound system (GE Healthcare, Chicago, IL, USA). Coronary diastolic flow velocities were recorded at rest and during pharmacologically induced hyperemia with intravenous adenosine (140 µg/kg/min). CFR was calculated as the ratio of hyperemic to baseline peak diastolic velocity. A prespecified cutoff of CFR <2.0 was used to define CMD, in accordance with international consensus statements and validation studies demonstrating that this threshold reflects impaired microvascular vasodilator capacity and is prognostically significant across TTDE, positron emission tomography (PET), and invasive studies [15-17].

Blinding, reproducibility, and quality control

All TTDE studies were performed by two experienced cardiologists blinded to patients’ clinical characteristics and outcomes, following a standardized protocol. Inter- and intra-observer reproducibility was assessed in a random 10% subset, demonstrating excellent agreement (intraclass correlation coefficient >0.80 for CFR). To ensure data quality, we implemented quarterly audits, 10% random chart re-abstraction, and automated range/consistency checks in the study database.

Follow-up and outcome adjudication

Patients were prospectively followed for 24 months, a clinically meaningful interval consistent with prior CMD studies showing that adverse cardiovascular outcomes in diabetic cohorts typically emerge within two to four years [13,18]. Follow-up included outpatient visits, structured telephone interviews, and review of electronic health records. Cardiovascular outcomes (myocardial infarction, heart failure hospitalization, and cardiovascular death) were adjudicated independently by two blinded physicians, with disagreements resolved by a third adjudicator.

Of 264 patients, 243 (CMD: 128; non-CMD: 115) completed follow-up; 21 (7.95%) were lost. Baseline characteristics of those lost did not differ significantly from retained patients (age, sex, diabetes duration, hypertension, smoking, HbA1c, BMI; all p>0.10), minimizing attrition bias.

Statistical analysis

Analyses were performed using IBM SPSS v26 (IBM Corp., Armonk, NY, USA) and verified in R (R Foundation for Statistical Computing, Vienna, Austria). Continuous variables are reported as mean ± SD, categorical variables as counts (%). Between-group comparisons used Student’s t-test or Chi-square tests as appropriate. Survival analyses used Kaplan-Meier curves with log-rank testing. To address confounding, we fitted multivariable Cox proportional hazards models with CMD (CFR <2.0) as the primary exposure, adjusting for age, sex, hypertension, dyslipidemia, BMI, diabetes duration, HbA1c, smoking status, and baseline medications (statins, ACEi/ARB, beta-blockers, SGLT2 inhibitors, insulin). Proportional hazards assumptions were verified using Schoenfeld residuals.

Missing baseline covariates (<5% per variable) were imputed using multiple imputation by chained equations (m=10), with results concordant with complete-case analyses. Patients lost to follow-up were excluded from time-to-event analyses, and all denominators (baseline 142 vs. 122; analyzed 128 vs. 115) are reported transparently. A two-sided p<0.05 was considered statistically significant, and hazard ratios (HR) with 95% confidence intervals (CI) are presented. We additionally performed subgroup and sensitivity analyses stratified by baseline statin use, insulin therapy, and HbA1c quartiles to explore treatment-outcome interactions. Variance inflation factors (VIF) were <2 for all covariates, excluding multicollinearity.

Bias-reduction strategies included systematic exclusion of obstructive CAD using angiography or CTCA in all patients (to isolate CMD-specific effects); Blinding of operators and outcome adjudicators to clinical data and CFR results; Assessment of inter-/intra-observer reproducibility for CFR; Multiple imputation for missing baseline covariates (<5%), ensuring robustness; Comparison of baseline characteristics of patients lost to follow-up with those retained, showing no significant differences, thus minimizing attrition bias and sensitivity analyses (complete-case vs. imputed datasets) yielding concordant results.

Patients lost to follow-up were excluded from time-to-event analyses, and all denominators (baseline 142 vs. 122; analyzed 128 vs. 115) are reported transparently. A two-sided p<0.05 was considered statistically significant, and HR with 95% CI are reported.

Ethical approval

The study protocol was reviewed and approved by the Institutional Review Board (approval EC-508-22). Written informed consent was obtained from all participants prior to enrollment. Patient confidentiality was maintained in accordance with the ethical principles outlined in the Declaration of Helsinki.

## Results

Among the 264 enrolled patients, 142 (53.79%) had CMD (CFR < 2.0) and 122 (46.21%) did not (Table 1). The CMD group was older (59.45 ± 8.12 vs. 56.28 ± 7.97 years, p=0.002) and had a longer duration of diabetes (12.35 ± 6.21 vs. 9.87 ± 5.43 years, p<0.001). Hypertension was more prevalent in the CMD group (66.90% vs. 53.28%, p=0.03), and the CMD group also had a higher mean BMI (28.12 ± 4.03 vs. 26.78 ± 3.74 kg/m², p=0.005). Gender distribution and smoking history were similar between groups.

**Table 1:** Baseline Demographic and Clinical Characteristics of the Study Population (N = 264) CMD: coronary microvascular dysfunction * P-values are significant (<0.05)

Category	Characteristic	CMD Present (n = 142)	CMD Absent (n = 122)	Mean/Proportion Difference (95% CI)	Test Used	Test Statistic	p-value
Demographic Characteristics	Age, years (mean ± SD)	59.45 ± 8.12	56.28 ± 7.97	+3.17 (1.19–5.15)	t-test	3.19	0.002*
	Male, n (%)	82 (57.75)	68 (55.74)	–	Chi-square	0.04	0.841
	Female, n (%)	60 (42.25)	54 (44.26)	–	Chi-square	0.04	0.841
Clinical History	Duration of Diabetes (years)	12.35 ± 6.21	9.87 ± 5.43	+2.48 (1.06–3.90)	t-test	3.43	<0.001*
	Hypertension, n (%)	95 (66.90)	65 (53.28)	–	Chi-square	4.55	0.033*
	Smoking history, n (%)	45 (31.69)	30 (24.59)	–	Chi-square	1.30	0.254
Anthropometric Measures	Body Mass Index (kg/m²)	28.12 ± 4.03	26.78 ± 3.74	+1.34 (0.40–2.29)	t-test	2.78	0.006*

Patients with CMD had significantly worse metabolic and echocardiographic profiles at baseline (Table 2). Mean HbA1c was higher in the CMD group (8.75 ± 1.45% vs. 7.89 ± 1.31%, p<0.001), along with elevated low-density lipoprotein (LDL) cholesterol (118.54 ± 29.72 vs. 109.23 ± 26.48 mg/dL, p=0.01) and triglycerides (172.54 ± 64.88 vs. 150.67 ± 58.13 mg/dL, p=0.02). Conversely, high-density lipoprotein (HDL) cholesterol was lower (38.45 ± 9.10 vs. 42.12 ± 10.05 mg/dL, p=0.002). Hemodynamically, CMD patients had higher systolic and diastolic blood pressures (138.90 ± 17.02/82.13 ± 9.45 vs. 132.45 ± 15.78/79.57 ± 8.99 mmHg, p=0.002 and p=0.03, respectively). Left ventricular ejection fraction was modestly reduced (58.74 ± 5.11% vs. 60.18 ± 4.85%, p=0.01). Most notably, CFR was profoundly impaired in CMD patients (1.69 ± 0.22 vs. 2.54 ± 0.38, p<0.001), confirming the presence of significant coronary microvascular dysfunction.

**Table 2:** Laboratory and Echocardiographic Parameters at Baseline CMD: coronary microvascular dysfunction; SD: standard deviation; BP: blood pressure; LVEF: left ventricular ejection fraction; HDL: high-density lipoprotein; LDL: low-density lipoprotein; SBP: systolic blood pressure; DBP: diastolic blood pressure; CFR: coronary flow reserve Test Used: Independent samples t-test was applied for continuous variables *p-values < 0.05 are considered statistically significant

Parameter	CMD Present (n=142)	CMD Absent (n=122)	Mean Difference (95% CI)	p-value
HbA1c (%)	8.75 ± 1.45	7.89 ± 1.31	+0.86 (0.51–1.21)	<0.001*
LDL (mg/dL)	118.54 ± 29.72	109.23 ± 26.48	+9.31 (2.27–16.35)	0.01*
HDL (mg/dL)	38.45 ± 9.10	42.12 ± 10.05	–3.67 (–6.00 to –1.34)	0.002*
Triglycerides (mg/dL)	172.54 ± 64.88	150.67 ± 58.13	+21.87 (4.23–39.51)	0.02*
SBP (mmHg)	138.90 ± 17.02	132.45 ± 15.78	+6.45 (2.40–10.50)	0.002*
DBP (mmHg)	82.13 ± 9.45	79.57 ± 8.99	+2.56 (0.21–4.91)	0.03*
LVEF (%)	58.74 ± 5.11	60.18 ± 4.85	–1.44 (–2.59 to –0.29)	0.01*
CFR	1.69 ± 0.22	2.54 ± 0.38	–0.85 (–0.92 to –0.78)	<0.001*

When baseline parameters were analyzed in relation to the composite end-point (acute myocardial infarction, heart failure hospitalization, and all-cause death), CFR was the strongest predictor of risk. Each 0.1-unit decrease in CFR conferred a 12% increase in hazard (HR 1.12, 95% CI 1.07-1.17, p<0.001), and CFR <2.0 nearly doubled the risk (HR 2.41, 95% CI 1.39-4.16, p=0.002). Among metabolic factors, HbA1c per 1% increase (HR 1.21, 95% CI 1.08-1.35, p=0.001) and LDL cholesterol per 10 mg/dL increase (HR 1.07, 95% CI 1.01-1.14, p=0.029) were independently associated with worse outcomes, while HDL cholesterol per 5 mg/dL increase was protective (HR 0.90, 95% CI 0.83-0.98, p=0.017). Higher systolic blood pressure (BP) (per 10 mmHg, HR 1.15, 95% CI 1.03-1.28, p=0.013) and lower LVEF (per 5%, HR 0.88, 95% CI 0.79-0.98, p=0.022) also contributed to prognosis. Triglycerides showed a borderline trend (HR 1.08 per 50 mg/dL, 95% CI 0.99-1.18, p=0.077), while diastolic BP was not independently significant. Importantly, adding CFR to a base model of traditional risk factors significantly improved prognostic discrimination (likelihood-ratio test p<0.001), underscoring its incremental value in risk stratification.

Baseline medication use, including antidiabetic, antihypertensive, lipid-lowering, and antiplatelet therapies, was broadly comparable between CMD and non-CMD groups (Table 3). Metformin was the most commonly prescribed antidiabetic agent in both groups (n=112, 78.9% vs. n=97, 79.5%), but it was not the only therapy used. Several patients were also on insulin, sulfonylureas, dipeptidyl peptidase 4 (DPP-4) inhibitors, and SGLT2 inhibitors, reflecting the progressive and multifaceted treatment needs of type 2 diabetes. Insulin therapy was more frequently used in the CMD group (n=60, 42.3%) compared to the non-CMD group (n=41, 33.6%). With respect to lipid-lowering therapy, statins were prescribed in the majority but not all patients-63.4% (n=90) with CMD and 54.9% (n=67) without CMD - consistent with real-world practice where statin use may vary based on baseline lipid profile, overall cardiovascular risk, tolerance, and physician judgment. Similarly, ACE inhibitors or ARBs were used in 59.9% of CMD patients (n=85) and 56.6% of non-CMD patients (n=69), while beta-blocker use was nearly identical across groups (38.0% vs. 36.9%).

**Table 3:** Medication Use at Baseline (N = 264) CMD: coronary microvascular dysfunction; ACEi: angiotensin-converting enzyme inhibitor; ARB: angiotensin receptor blocker; SGLT2i: sodium-glucose cotransporter-2 inhibitor; DPP-4i: dipeptidyl peptidase-4 inhibitor

Medication Category	CMD Present (n=142)	CMD Absent (n=122)	χ²-value	p-value
Metformin, n (%)	112 (78.9)	97 (79.5)	0.01	0.91
Insulin, n (%)	60 (42.3)	41 (33.6)	2.16	0.14
Statins, n (%)	90 (63.4)	67 (54.9)	1.78	0.18
ACE inhibitors/ARBs, n (%)	85 (59.9)	69 (56.6)	0.23	0.63
Beta-blockers, n (%)	54 (38.0)	45 (36.9)	0.03	0.87
SGLT2 inhibitors, n (%)	12 (8.5)	10 (8.2)	0.01	0.94
DPP-4 inhibitors, n (%)	15 (10.6)	13 (10.7)	0.00	0.98
Sulfonylureas, n (%)	18 (12.7)	14 (11.5)	0.08	0.78
Antiplatelets, n (%)	40 (28.2)	32 (26.2)	0.12	0.73

**Figure 1:**
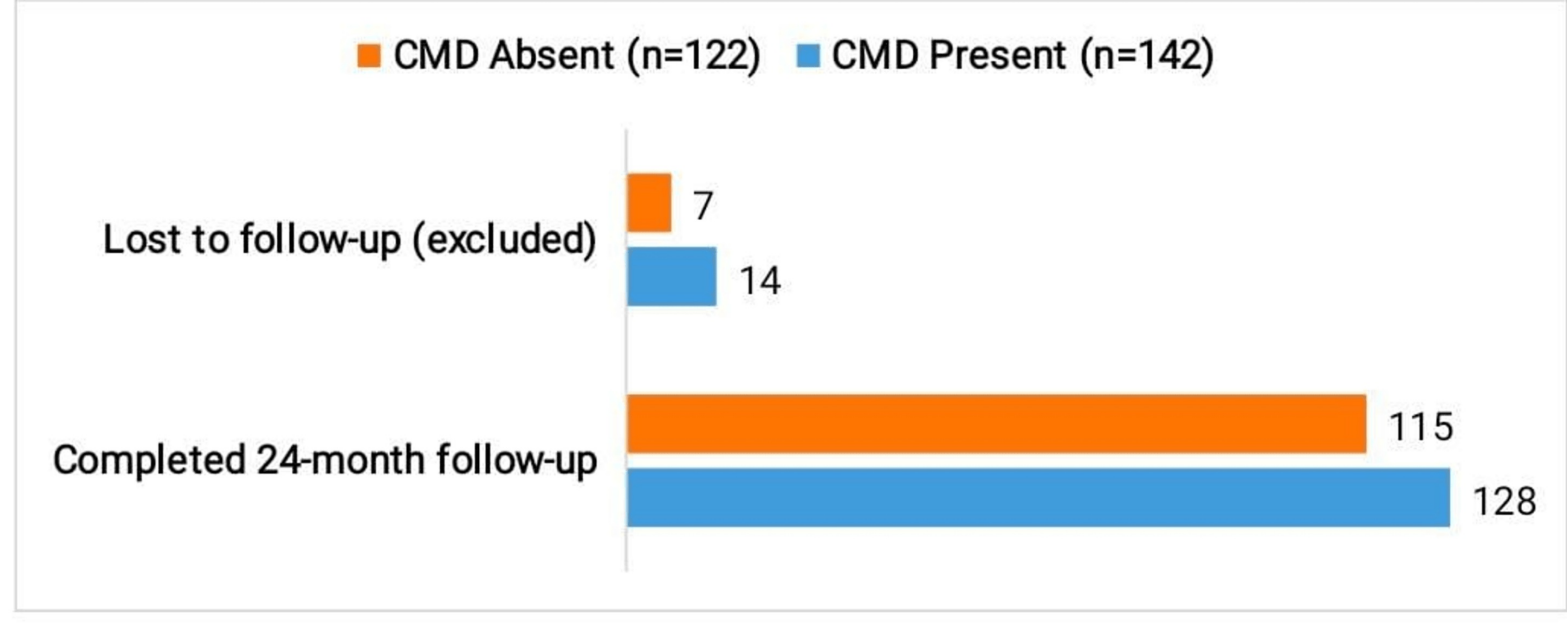
Follow-up Status CMD: coronary microvascular dysfunction

Of the total, 243 patients (128 with CMD and 115 without CMD) completed the 24-month follow-up, with follow-up completion rates of 90.14% in the CMD group and 94.26% in the non-CMD group (Figure 1). A total of 21 patients (7.95%) were lost to follow-up and excluded from time-to-event analysis.

Patients with CMD experienced significantly higher rates of MACE, occurring in 38 out of 128 patients (29.69%) compared to 12 out of 115 patients (10.43%) in the non-CMD group (p<0.001), shown in Table 4. Myocardial infarction was reported in 15 patients (11.72%) with CMD versus five patients (4.35%) without CMD (p=0.03). Hospitalization for heart failure occurred in 22 CMD patients (17.19%) compared to six non-CMD patients (5.22%) (p=0.004). All-cause mortality was also significantly higher among CMD patients, with 18 deaths (14.06%) compared to seven deaths (6.09%) in the non-CMD group (p=0.04). Cardiovascular death showed a non-significant trend toward higher occurrence in the CMD group, affecting 10 patients (7.81%) versus three patients (2.61%) in the non-CMD group (p=0.06).

**Table 4:** Long-Term Cardiovascular Outcomes Over 24 Months (Based on Followed-Up Patients Only) CMD: coronary microvascular dysfunction Chi-square test was used to compare proportions between groups * p-values < 0.05 are considered statistically significant

Outcome	CMD Present (n=128)	CMD Absent (n=115)	χ²-value	p-value	Hazard Ratio (HR)	95% CI
Major Adverse Cardiovascular Events (MACE), n (%)	38 (29.7)	12 (10.4)	13.82	<0.001*	2.41	1.39–4.16
Myocardial Infarction, n (%)	15 (11.7)	5 (4.4)	4.76	0.03*	2.28	1.01–5.16
Heart Failure Hospitalization, n (%)	22 (17.2)	6 (5.2)	8.27	0.004*	2.85	1.19–6.80
Cardiovascular Death, n (%)	10 (7.8)	3 (2.6)	3.45	0.06	2.94	0.82–10.53
All-Cause Mortality, n (%)	18 (14.1)	7 (6.1)	4.18	0.04*	2.34	1.01–5.42z

**Figure 2:**
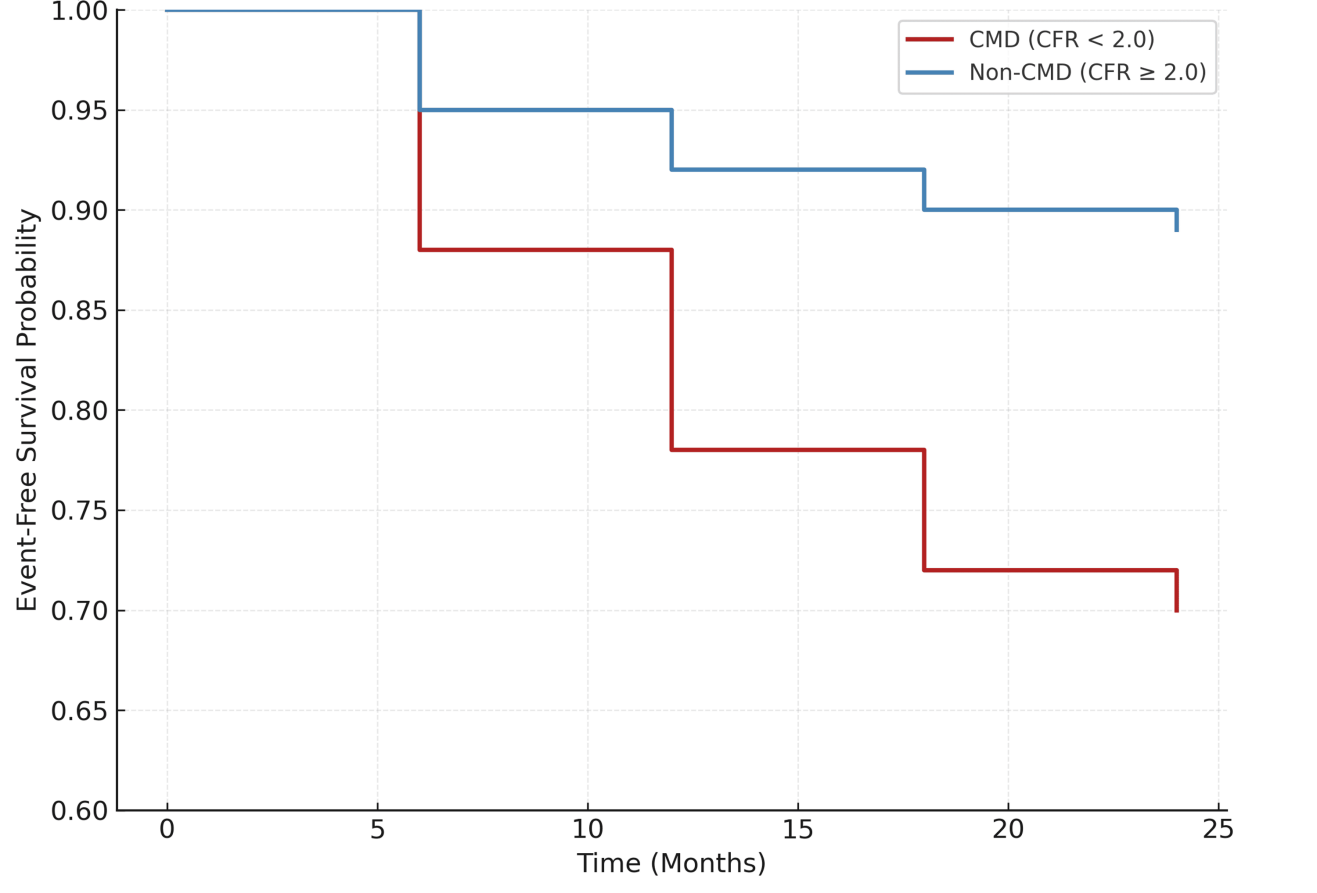
Kaplan–Meier survival curves for MACE, visually comparing event-free survival between CMD (CFR <2.0) and non-CMD (CFR ≥2.0) groups over 24 months. CMD: coronary microvascular dysfunction; MACE: major adverse cardiovascular events; CFR: coronary flow reserve

Figure 2 shows Kaplan-Meier survival curves for MACE over 24 months, demonstrating significantly lower event-free survival in patients with CMD (CFR <2.0) compared to those without CMD (CFR ≥2.0), highlighting the strong prognostic impact of impaired coronary microvascular function in type 2 diabetes.

Kaplan-Meier analysis (Table 5) showed significantly shorter median event-free survival in the CMD group for MACE (19.5 vs. >24 months, p<0.001), myocardial infarction (21.3 vs. >24 months, p=0.029), and heart failure hospitalization (20.7 vs. >24 months, p=0.004), shown in Table 4. Although cardiovascular death showed a trend (22.8 vs. >24 months, p=0.063), all-cause mortality was significantly higher in CMD patients (21.6 vs. >24 months, p=0.041).

**Table 5:** Kaplan–Meier Survival Estimates and Log-Rank Test Results for Cardiovascular Outcomes. CMD: coronary microvascular dysfunction *p < 0.05 considered statistically significant

Outcome	Event Rate CMD Present	Event Rate CMD Absent	Median Event-Free Survival (Months)	Log-Rank χ²	p-value	Hazard Ratio (HR)	95% CI
Major Adverse Cardiovascular Events (MACE)	29.7%	10.4%	CMD: 19.5 vs. Non-CMD: >24	13.82	<0.001*	2.41	1.39–4.16
Myocardial Infarction	11.7%	4.4%	CMD: 21.3 vs. Non-CMD: >24	4.76	0.029*	2.28	1.01–5.16
Heart Failure Hospitalization	17.2%	5.2%	CMD: 20.7 vs. Non-CMD: >24	8.27	0.004*	2.85	1.19–6.80
Cardiovascular Death	7.8%	2.6%	CMD: 22.8 vs. Non-CMD: >24	3.45	0.063	2.94	0.82–10.53
All-Cause Mortality	14.1%	6.1%	CMD: 21.6 vs. Non-CMD: >24	4.18	0.041*	2.34	1.01–5.42

## Discussion

In this prospective T2DM cohort without obstructive CAD, CFR measured by TTDE - an absolute, physiology-based index of microvascular function - was the most powerful predictor of the composite end-point, even after adjustment for traditional risk factors, metabolic profile, left ventricular (LV) systolic function, and cardio-metabolic therapies. The graded, continuous relation (per 0.1 unit decrease) indicates that risk increases well before the conventional <2.0 threshold and supports the biological plausibility that progressive microvascular dysfunction drives ischemia, heart failure decompensation, and mortality in diabetes. Importantly, our findings align with prior interventional evidence suggesting that therapies may favorably influence CMD: for instance, liraglutide improved weight and microvascular function in women with CMD [19], while comprehensive treatment strategies addressing weight, BP, and endothelial function improved microvascular angina outcomes [20]. These trials highlight that CMD is potentially modifiable, and our results reinforce the need for targeted interventions in diabetics with impaired CFR.

The independent associations of higher HbA1c, higher SBP, and higher LDL cholesterol with events underscore the synergistic impact of poor glycemic control, suboptimal BP, and atherogenic lipids on microvascular integrity and downstream outcomes, whereas higher HDL cholesterol and higher LVEF were protective. Importantly, CFR retained significance after adjustment for these factors and for baseline therapies (statins, ACEi/ARB, SGLT2 inhibitors, insulin), highlighting microvascular dysfunction as an independent prognostic pathway not fully captured by risk-factor control alone. These findings align with prior literature showing CFR’s prognostic value in diabetics and mixed populations and extend it by demonstrating incremental value in a resource-constrained, LMIC setting using TTDE [21-26].

This research shows that there is a strong link between CMD and worse long-term heart health outcomes in people with type 2 diabetes. Out of the 264 patients who signed up, 53.79% were diagnosed with CMD based on a CFR of less than 2.0, which is in line with previous research that reported CMD as common in people with diabetes due to persistent metabolic dysregulation [21]. Patients with CMD had a poorer cardiometabolic profile, with higher HbA1c (8.75 ± 1.45% vs. 7.89 ± 1.31%, p<0.001), higher LDL (118.54 ± 29.72 vs. 109.23 ± 26.48 mg/dL, p=0.01), and lower HDL (38.45 ± 9.10 vs. 42.12 ± 10.05 mg/dL, p=0.002). These results are consistent with earlier investigations that found comparable metabolic risk factors in individuals with impaired CFR, strengthening the relationship between dyslipidemia, glycemic load, and CMD pathogenesis [22].

During the 24-month follow-up, 29.69% of CMD patients had MACE, whereas only 10.43% of non-CMD patients had them (p<0.001). The CMD group also had a greater likelihood of myocardial infarction (MI) (11.72% vs. 4.35%, p=0.03). This is consistent with prior studies showing that individuals with lower CFR had a more than two-fold higher risk of MI, even without significant epicardial disease [23]. Additionally, CMD patients were more likely to be hospitalized for heart failure (17.19% vs. 5.22%, p=0.004), supporting the concept that microvascular ischemia contributes to myocardial remodeling and diastolic dysfunction, particularly in diabetic cardiomyopathy [24].

Our results also demonstrate that CMD patients had a higher risk of dying from all causes (14.06% vs. 6.09%, p=0.04). This aligns with previous studies that showed CMD to be an independent predictor of mortality in both diabetic and non-diabetic populations [25]. While cardiovascular death only showed a trend toward significance (7.81% vs. 2.61%, p=0.06), the relatively small sample size and limited number of cardiovascular deaths likely reduced statistical power rather than indicating a true absence of effect. Kaplan-Meier survival analysis confirmed shorter event-free survival in CMD patients for MACE (median 19.5 vs. >24 months, p<0.001), MI (21.3 vs. >24 months, p=0.029), and heart failure (20.7 vs. >24 months, p=0.004), in line with temporal trends reported by other cohorts examining CFR impairment [13,18,26].

Study strengths and limitations

This study provides valuable insights into how CMD impacts prognosis in diabetes, particularly in a low- to middle-income country setting where such data are scarce. Its strengths include a prospective design, use of TTDE to non-invasively measure CFR, and 24-month follow-up. However, limitations include single-center convenience sampling (raising referral bias concerns), reliance on TTDE rather than PET or CMR, lack of a formal sample size calculation, and exclusion of patients lost to follow-up, which may have underestimated event rates. CMD may also partly act as a marker of overall disease burden rather than a purely independent driver of events; further multicenter and mechanistic studies are needed to disentangle causality and test whether interventions targeting CMD can improve outcomes.

## Conclusions

In this study, CMD was significantly associated with a higher risk of major adverse cardiovascular events-including myocardial infarction and heart failure hospitalization-in patients with type 2 diabetes without obstructive CAD. While these findings support the prognostic importance of CMD, they should be interpreted as associations and not definitive proof of causality. Unmeasured confounders may also have contributed.

Across baseline parameters, absolute CFR measured by TTDE was the strongest, independent prognostic factor for the composite of acute myocardial infarction, heart failure admission, and all-cause death. Additional risk was conferred by higher HbA1c, systolic blood pressure, and LDL cholesterol, whereas higher HDL cholesterol and preserved LVEF were protective. These findings support a dual approach: integrating CFR assessment into cardiovascular risk stratification, while simultaneously ensuring aggressive optimization of glycemia, blood pressure, and lipids.

Further large-scale and interventional studies are needed to confirm whether CMD represents not only a marker of disease burden but also a mechanistic driver of poor outcomes. Early recognition and management of CMD - including intensive risk factor modification and the use of cardioprotective agents such as SGLT2 inhibitors and GLP-1 receptor agonists - may improve prognosis. However, wider implementation is constrained by diagnostic availability and cost, highlighting the need for accessible, practical tools for CMD detection in routine practice.

## References

[1] Balakumar P, Maung-U K, Jagadeesh G (2016). Prevalence and prevention of cardiovascular disease and diabetes mellitus. Pharmacol Res.

[2] Arokiasamy P, Salvi S, Selvamani Y (2021). Global burden of diabetes mellitus. Handbook of Global Health.

[3] Tognola C, Maloberti A, Varrenti M, Mazzone P, Giannattasio C, Guarracini F (2025). Myocardial infarction with nonobstructive coronary arteries (MINOCA): current insights into pathophysiology, diagnosis, and management. Diagnostics (Basel).

[4] Crea F, Montone RA, Rinaldi R (2022). Pathophysiology of coronary microvascular dysfunction. Circ J.

[5] Lanza GA, Crea F (2010). Primary coronary microvascular dysfunction: clinical presentation, pathophysiology, and management. Circulation.

[6] Vancheri F, Longo G, Vancheri S, Henein M (2020). Coronary microvascular dysfunction. J Clin Med.

[7] Salvatore T, Galiero R, Caturano A (2022). Coronary microvascular dysfunction in diabetes mellitus: pathogenetic mechanisms and potential therapeutic options. Biomedicines.

[8] Singh A, Ashraf S, Irfan H (2025). Heart failure and microvascular dysfunction: an in-depth review of mechanisms, diagnostic strategies, and innovative therapies. Ann Med Surg (Lond).

[9] D'Amario D, Migliaro S, Borovac JA (2019). Microvascular dysfunction in heart failure with preserved ejection fraction. Front Physiol.

[10] Ong P, Safdar B, Seitz A, Hubert A, Beltrame JF, Prescott E (2020). Diagnosis of coronary microvascular dysfunction in the clinic. Cardiovasc Res.

[11] Al Badarin F, Aljizeeri A, Almasoudi F, Al-Mallah MH (2017). Assessment of myocardial blood flow and coronary flow reserve with positron emission tomography in ischemic heart disease: current state and future directions. Heart Fail Rev.

[12] Chowdhary A, Garg P, Das A, Nazir MS, Plein S (2021). Cardiovascular magnetic resonance imaging: emerging techniques and applications. Heart.

[13] Cortigiani L, Rigo F, Gherardi S, Sicari R, Galderisi M, Bovenzi F, Picano E (2007). Additional prognostic value of coronary flow reserve in diabetic and nondiabetic patients with negative dipyridamole stress echocardiography by wall motion criteria. J Am Coll Cardiol.

[14] Kelshiker MA, Seligman H, Howard JP (2022). Coronary flow reserve and cardiovascular outcomes: a systematic review and meta-analysis. Eur Heart J.

[15] Chen C, Wei J, AlBadri A, Zarrini P, Bairey Merz CN (2016). Coronary microvascular dysfunction―epidemiology, pathogenesis, prognosis, diagnosis, risk factors and therapy. Circ J.

[16] Lukose T, Sood A, Mittal B, Barwad P, Dutta P (2019). Evaluation of coronary microvascular dysfunction in long-standing asymptomatic diabetic patients with 13N-NH3 cardiac PET. J Nucl Med.

[17] Leung M, Leung DY (2016). Coronary microvascular function in patients with type 2 diabetes mellitus. EuroIntervention.

[18] Murthy VL, Naya M, Foster CR (2011). Improved cardiac risk assessment with noninvasive measures of coronary flow reserve. Circulation.

[19] Suhrs HE, Raft KF, Bové K, Madsbad S, Holst JJ, Zander M, Prescott E (2019). Effect of liraglutide on body weight and microvascular function in non-diabetic overweight women with coronary microvascular dysfunction. Int J Cardiol.

[20] Bove KB, Nilsson M, Pedersen LR, Mikkelsen N, Suhrs HE, Astrup A, Prescott E (2020). Comprehensive treatment of microvascular angina in overweight women - a randomized controlled pilot trial. PLoS One.

[21] Kibel A, Selthofer-Relatic K, Drenjancevic I, Bacun T, Bosnjak I, Kibel D, Gros M (2017). Coronary microvascular dysfunction in diabetes mellitus. J Int Med Res.

[22] Horton WB, Barrett EJ (2021). Microvascular dysfunction in diabetes mellitus and cardiometabolic disease. Endocr Rev.

[23] Majmudar MD, Murthy VL, Shah RV (2015). Quantification of coronary flow reserve in patients with ischaemic and non-ischaemic cardiomyopathy and its association with clinical outcomes. Eur Heart J Cardiovasc Imaging.

[24] Rush CJ, Berry C, Oldroyd KG (2021). Prevalence of coronary artery disease and coronary microvascular dysfunction in patients with heart failure with preserved ejection fraction. JAMA Cardiol.

[25] Zhang W, Singh S, Liu L (2022). Prognostic value of coronary microvascular dysfunction assessed by coronary angiography-derived index of microcirculatory resistance in diabetic patients with chronic coronary syndrome. Cardiovasc Diabetol.

[26] Gurgoglione FL, Benatti G, Denegri A (2025). Coronary microvascular dysfunction: insights on prognosis and future perspectives. Rev Cardiovasc Med.

